# A semisynthetic Atg3 reveals that acetylation promotes Atg3 membrane binding and Atg8 lipidation

**DOI:** 10.1038/ncomms14846

**Published:** 2017-03-22

**Authors:** Yi-Tong Li, Cong Yi, Chen-Chen Chen, Huan Lan, Man Pan, Shao-Jin Zhang, Yi-Chao Huang, Chao-Jian Guan, Yi-Ming Li, Li Yu, Lei Liu

**Affiliations:** 1 School of Biological and Medical Engineering, Hefei University of Technology, Anhui, Hefei 230009, China; 2 Tsinghua-Peking Center for Life Sciences, Key Laboratory of Bioorganic Phosphorus Chemistry & Chemical Biology (Ministry of Education), Department of Chemistry, Tsinghua University, Beijing 100084, China; 3 Tsinghua-Peking Center for Life Sciences, Key Laboratory of Biomembrane and Membrane Biotechnology, Center for Life Sciences, School of Life Sciences, Tsinghua University, Beijing 100084, China

## Abstract

Acetylation of Atg3 regulates the lipidation of the protein Atg8 in autophagy. The molecular mechanism behind this important biochemical event remains to be elucidated. We describe the first semi-synthesis of homogeneous K19/K48-diacetylated Atg3 through sequential hydrazide-based native chemical ligation. *In vitro* reconstitution experiments with the semi-synthetic proteins confirm that Atg3 acetylation can promote the lipidation of Atg8. We find that acetylation of Atg3 enhances its binding to phosphatidylethanolamine-containing liposomes and to endoplasmic reticulum, through which it promotes the lipidation process.

## Abstract (web summary)

Acetylation of Atg3 regulates lipidation of Atg8 and therefore autophagy, but the molecular mechanisms remain unclear. Here, Li *et al*. semi-synthesize diacetylated Atg3, allowing them to show that acetylated Atg3 enhances Atg8 lipidation by promoting interaction of Atg3 with liposomes containing physiological levels of phosphatidylethanolamine.

Autophagy is a catabolic process for the degradation of cellular contents^1^. One of the key biochemical events of autophagy is the lipidation of protein Atg8 with phosphatidylethanolamine (PE) by a series of enzymatic reactions^2^. In yeast, the lipidation reaction is catalysed by two ubiquitin-like pathways. First, Atg7, an E1-like enzyme activates Atg8 and the activated Atg8 is transferred to an E2-like enzyme Atg3, with the help of Atg5-Atg12, which serves as an E3-like enzyme. Subsequently, Atg8 is conjugated with PE^3^. Atg8 lipidation has been successfully reconstituted *in vitro*, which has been intensively studied for dissecting molecular mechanism for autophagy regulation^4^. Previous studies showed that PE concentration was one of the key factor for determining lipidation efficiency. When PE concentration is high, the lipidation reaction can occur rapidly without Atg5-Atg12. However, under physiological concentration of PE, which ranges from 15 to 25%, the lipidation occurs very slowly without the presence of Atg5-Atg12 (ref. 5).

Protein acetylation emerges as an important regulatory mechanism for varies biological processes including glycolysis and autophagy^6^. It has been generally believed that acetylation carried out its function through two main mechanisms, by modulating protein–protein interaction or by competing with other modification such as ubiquitination^7^. We recently reported that acetylation of Atg3 regulated the strength and duration of autophagy. Specifically, two Lys residues, that is, K19 and K48 of Atg3 were acetylated by acetyltransferase Esa1, while removal of Atg3 acetylation by mutated K19/K48, or by damaging acetyltransferase activity of Esa1, led to markedly reduced autophagy level^8^. Although we observed the impaired Atg8 lipidation in these mutant cells, how Atg3 acetylation regulated Atg8 lipidation remained unknown. The multiple modification sites on Atg3 hampered our previous effort to accurately illustrate the mechanism.

Protein semi-synthesis is a powerful way to study the function and structure of proteins with post-translational modifications^9^. This strategy which combines the power of chemical peptide synthesis and recombinant protein expression affords unique advantages to generate proteins with site-specifically single or multiple post-translational modifications^10^. Protein semi-synthesis also enables the preparation of homogeneous forms of modified proteins used for performing detailed biophysical, biochemical and even X-ray crystallization studies^11^,^12^,^13^.

Here we report that acetylation regulates Atg8 lipidation by promoting the interaction between Atg3 and PE-containing liposome. K19/K48-diacetylated Atg3 protein (Atg3 K19ac-K48ac), which was free from other modifications, were semi-synthesized by using of our recently developed hydrazide-based native chemical ligation approach^14^,^15^,^16^. Biochemical assays with the semi-synthetic proteins confirmed that Atg3 acetylation could prompt the lipidation of Atg8 *in vitro*. Interestingly, we found that acetylation of Atg3 significantly enhanced the binding between Atg3 and liposome containing physiological level of PE, while had no obvious effect on binding between Atg3 and liposome containing higher amount of PE. Therefore, acetylation of Atg3 may promote Atg8 lipidation by allowing the lipidation reaction to occur at physiological level of PE.

## Results

### Semi-synthesis of Atg3 K19ac-K48ac

**Figure 1:**
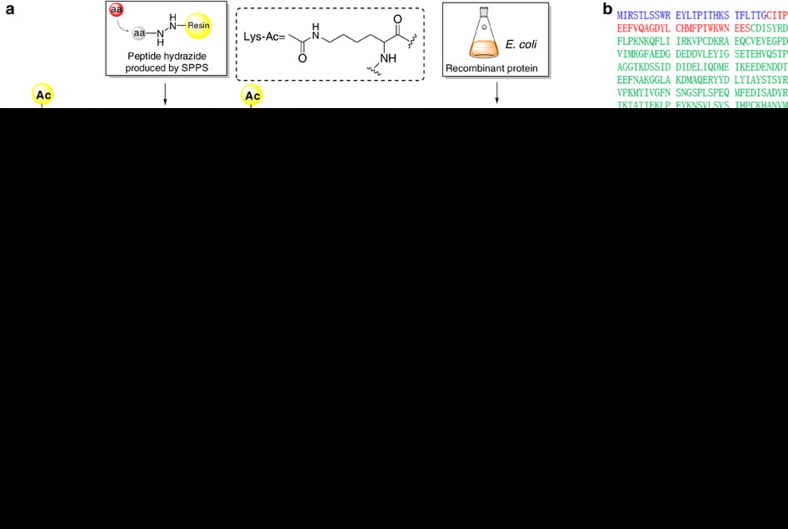
Chemical semi-synthesis of Atg3 K19ac-K48ac. (**a**) Schematic diagram of the synthetic route. (**b**) The amino-acid sequence of Atg3 with two cystine mutation. (**c**) Analytical HPLC chromatogram (*λ*=214 nm) and ESI-MS of 7. (**d**) SDS–PAGE analysis of Atg3 K19ac-K48ac. (**e**) Size exclusion chromatography showed a similar elution profile for refolded semi-synthetic Atg3 K19ac-K48ac and *E. coli* expressed WT Atg3. (**f**) CD spectra of Atg3 K19ac-K48ac and *E. coli* expressed WT Atg3.

Our study began with the semi-synthesis of the yeast *Saccharomyces cerevisiae* Atg3 K19ac-K48ac containing 310 amino acids (Fig. 1a,b). The Ser53-Ser54 bond in the N-terminal flexible loop was chosen as the ligation site^17^. Thus, Atg3(1-310) was divided into two segments, including chemically synthesized Atg3(1-53) peptide hydrazide and recombinant expressed Atg3(S54C-310), in which Ser54 was mutated to Cys54 for ligation. The acetylated Atg3(1-53) peptide hydrazide was initially synthesized through direct Fmoc (9-fluorenylmethoxycarbonyl) solid-phase peptide synthesis, but the product was difficult to purify from the crude peptide. To solve the problem, Atg3(1-53) was divided into two segments and the Gly26-Gln27 linkage in the N-terminal flexible loop was chosen as the ligation site with Gln27 mutated to Cys27 for ligation^17^. With this strategy, both the acetylated Atg3(1-26) and Atg3(27-53) peptide hydrazides were successfully generated (Supplementary Figs 1–2).

To prepare Atg3(54C-310), we first tried to express recombinant Atg3(54C-310) directly in *E. coli* BL21 (DE3) competence cells. Unfortunately, the N-terminal Cys residue of the expressed protein was blocked by formaldehyde to form a thiazolidine-derived byproduct^18^. Although we treated the thiazolidine-containing protein with methoxyamine at pH 4 to liberate the N-terminal Cys as reported in literature^19^, the efficiency was low (30% conversion on mass spectrum). To solve the problem, an enzymatically cleavable tag was fused in the N-terminal of the protein. In our initial attempt, GST-Atg3(54C-310) was expressed, purified with affinity chromatography and incubated with tobacco etch virus (TEV) protease at 4 °C overnight to excise glutathione S-transferase (GST) tag^20^. Unfortunately, the efficiency of this approach was still unsatisfactory (estimated to 30%), so that we turned to the SUMO tag fusion approach^21^. A His_6_-SUMO-Atg3(54C-310) fusion protein was overexpressed and purified on a Ni-NTA agarose resin. The His_6_-SUMO tag was then removed by the SUMO-specific protease with excellent efficiency (95% conversion within 4 h). Homogeneous Atg3(54C-310) was thus obtained through HPLC purification in a large quantity (Supplementary Fig. 3).

With three protein segments in hands, we carried out the semi-synthesis through sequential hydrazide-based native chemical ligation in the N to C direction^22^. First, Atg3(1-26)-NHNH_2_ (1.0 equiv.) dissolved in the aqueous phosphate (0.2 M) buffer containing 6.0 M guanidinium chloride (Gn·HCl) was oxidized by NaNO_2_ (10 equiv.) at pH 3.0, −15 °C for 15 min to produce a peptide acyl azide. MPAA (4-mercaptophenylacetic acid, 100 equiv.) was added and the pH value was adjusted to 6.5. After Atg3 (1-26)-NHNH_2_ was fully converted to a peptide thioester, Atg3(27-53)-NHNH_2_ (1.0 equiv.) was added to afford the ligation product Atg3(1-53)-NHNH_2_ in nearly quantitative conversion within 3 h (isolated yield=68%). Next, Atg3(1-53)-NHNH_2_ was ligated with the expressed protein segment Atg3(54C-310) in the same way to obtain the full-length peptide within 5 h (isolated yield=60%) (Supplementary Figs 4–5).

The full-length peptide was folded by gradient dialysis against decreasing urea concentration from 8 to 0 M^23^. After folding, Atg3 K19ac-K48ac was further purified by size exclusion chromatography, and the well-folded protein was collected in 70% yield at the same retention time as recombinant wild type (WT) Atg3 (Fig. 1e). HPLC, ESI-MS spectrum (Fig. 1c) and SDS–PAGE analysis (Fig. 1d) of the semi-synthetic Atg3 K19ac-K48ac also confirmed the right molecular weight and purity of the synthetic protein. The circular dichroism (CD) spectrum showed double negative peaks in the 200–230 nm region (Fig. 1f), which was almost identical as the recombinant WT sample, indicating correct folding. Furthermore, we prepared WT Atg3 without acetylation using the same semi-synthetic method (semi-synthetic Atg3 WT) as a control. This control protein was tested with both Atg8 lipidation and membrane-binding assays, confirming the validity of protein semi-synthesis (Supplementary Fig. 6).

### Reconstitution of Atg8 lipidation *in vitro*

**Figure 2:**
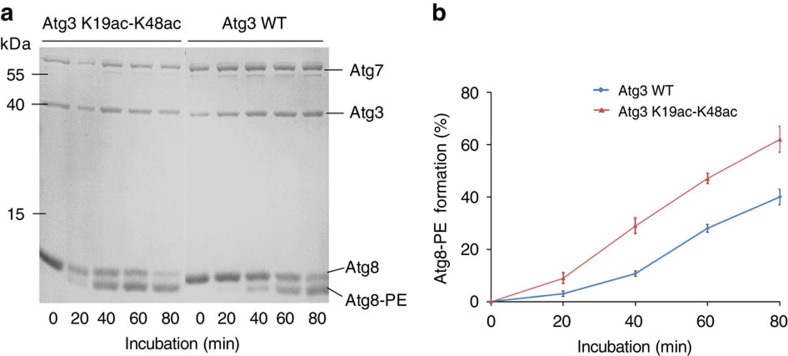
*In vitro* reconstitution of Atg8 lipidation. (**a**) WT Atg3 or Atg3 K19ac-K48ac (1 μM) was mixed with Atg7 (1 μM), Atg8 (10 μM), ATP (1 mM), PE-containing liposomes (350 μM) (200 nm liposomes composed of 10% bIPI, 20% DOPE and 70% POPC) and incubated at 30 °C for 80 min. SDS–PAGE analysis of reaction mixtures at different time intervals. (**b**) The efficiency of Atg8-PE formation catalysed by WT Atg3 (blue) and double acetylated Atg3 (red). The per cent was calculated by dividing the intensities of Atg8-PE by those of total Atg8 protein. The graph shows results of three independent experiments with error bars representing s.d.

Next, we aim to reconstitute the lipidation of Atg8 *in vitro*. Purified Atg7, Atg3, Atg8 and PE-containing liposomes were incubated together in the presence of ATP, and samples were taken from reaction mixture in 20 min intervals^24^. The collected samples were analysed by urea SDS–PAGE to examine the formation of Atg8-PE conjugates (Fig. 2a). To quantify the products (Atg8-PE conjugates), ChemiDoc software was used as previously described to monitor the progress of Atg8 lipidation *in vitro*^25^. As shown in Fig. 2b, the rate of Atg8-PE formation was significantly faster in the Atg3 K19ac-K48ac reaction system (yield=62±5% after 80 min incubation). When WT Atg3 was used in the lipidation process, the yield was only 40±3% after 80 min reaction.

To calculate the reaction rate constants for Atg3 WT and Atg3 K19ac-K48ac, we elongated the incubation time of Atg8 lipidation until 200 min. Both of the two reactions displayed hyperbolic curves for their progression and their rate constants were calculated using a pseudo-first-order reaction model. The reaction rate constant of Atg3 K19ac-K48ac was calculated to be 0.012 min^−1^, whereas the reaction rate constant of Atg3 WT was 0.0063, min^−1^ (Supplementary Fig. 7). These data confirmed that the reaction was accelerated by twofold when Atg3 K19-K48 was diacetylated. The same magnitude of reactivity increase was also observed in the *in vivo* experiments in yeast cells (Supplementary Fig. 8). Taken together, our *in vitro* experiments confirmed that K19ac-K48ac acetylation can promote the lipidation of Atg8.

### Effect of acetylation on Atg3 and Atg8 interaction

**Figure 3:**
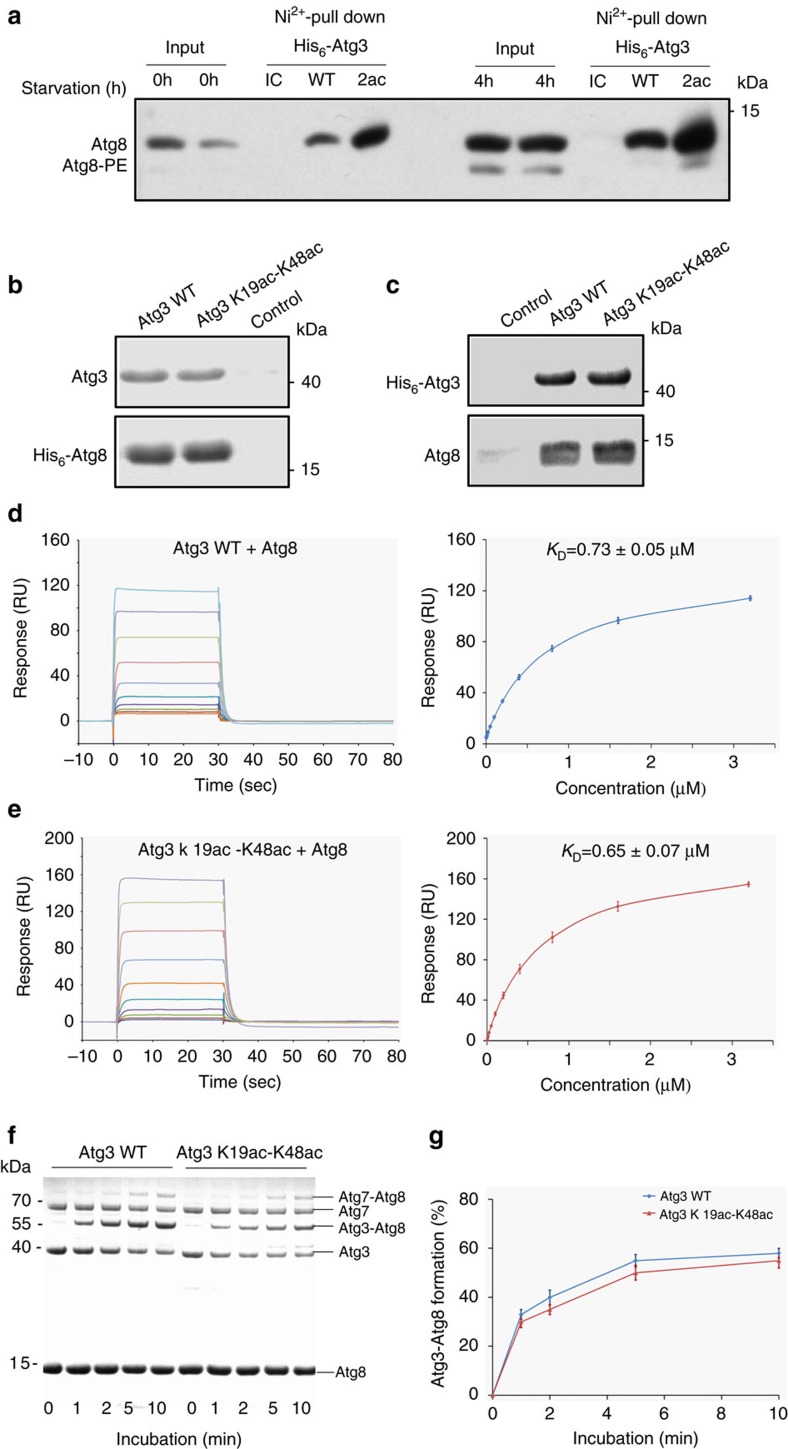
Interaction between Atg3 and Atg8 *in vitro*. (**a**) WT (BY4741) cells were starved in SD-N (nitrogen-free medium) for 0 and 4 h, and cell lysates were added His_6_-Atg3 or His_6_-Atg3 K19ac-K48ac-associated Ni^2+^ beads. The association of Atg3 with Atg8 was analysed by western blot by using the indicated antibodies. WT, Atg3 WT; 2ac, Atg3 K19ac-K48ac; IC, mouse IgG1 isotype control. (**b**) The bacterially expressed and purified His_6_-Atg8 was incubated with bacterially expressed and purified Atg3 WT or semi-synthetic Atg3 K19ac-K48ac, and bound proteins were detected by Coomassie Brilliant Blue staining. (**c**) The bacterially expressed His_6_-Atg3 WT or semi-synthetic His_6_-Atg3 K19ac-K48ac was incubated with the bacterially expressed and purified Atg8, and bound proteins were detected by Coomassie Brilliant Blue staining. C, control. (**d**) SPR binding studies of WT Atg3 to Atg8. (**e**) SPR binding studies of Atg3 K19ac-K48ac to Atg8. (**f**) Formation of Atg3-Atg8 thioester intermediate was analysed by SDS–PAGE. (**g**) Atg3-Atg8 thioester formation efficiency, calculated by dividing the intensities of Atg3-Atg8 thioester by those of total Atg3 protein. The graph shows results of three independent experiments and error bars represent s.d.

To understand how acetylation of Atg3 enhances Atg8-PE formation, we mixed His_6_-Atg3 WT or His_6_-Atg3 K19ac-K48ac with lysates from Atg8-expressing cells and then carried out the pull-down assay using Ni^2+^ beads. Consistent with our previous results, which showed that Atg3 acetylation is required for Atg3/Atg8 interaction, His_6_-Atg3 K19ac-K48ac pulls down significantly larger amount of Atg8 when compared to His-Atg3 WT (Fig. 3a).

Next, we examined whether acetylation can strengthen the interaction between Atg3 and Atg8 *in vitro*^8^. We used purified His_6_-Atg8 from *E. coli*, which is free of modification to perform a pull-down assay, and in this way, the purified His_6_-Atg3 WT and His_6_-Atg3 K19ac-K48ac were also used for bacterially expressed Atg8. We were surprised to find that Atg8 exhibited similar affinity for Atg3 K19ac-K48ac and WT Atg3 (Fig. 3b,c).

To quantify the effect of acetylation on the interaction between Atg3 and Atg8, surface plasmon resonance (SPR) (Biacore T200, at 25 °C) was used to measure the binding affinities. Thus, either Atg3 K19ac-K48ac or WT Atg3 was immobilized onto the surface of a CM5 sensor chip by amine coupling. Then Atg8 at different concentrations was flowed through the surface of the sensor chip. Our measured binding affinity (*K*_D_) between Atg3 K19ac-K48ac and Atg8 was 0.65±0.07 μM, while the *K*_D_ value between Atg3 WT and Atg8 was 0.73±0.05 μM (Fig. 3d,e). To corroborate the above data, we also used microscale thermophoresis (MST) to measure the binding affinity. With this technology, the *K*_D_ value for Atg3 K19ac-K48ac was 0.49±0.08 μM, whereas the *K*_D_ value for Atg3 WT was 0.52±0.07 μM (Supplementary Fig. 9). Taken together, our *in vitro* experiments showed that although acetylation of Atg3 enhances the interaction between Atg3 and Atg8 *in vivo*, the acetylation of Atg3 did not alter the interaction between Atg3 and Atg8 *in vitro*. Therefore, the increased Atg8 lipidation in Atg3 K19ac-K48ac should be attributed to other mechanism.

### Effect of acetylation on Atg3-Atg8 thioester formation

Next, we monitored the formation of the Atg3-Atg8 thioester intermediate in the lipidation process. Thus, Atg7, Atg3 (either WT or acetylated one) and Atg8 were incubated with ATP in the absence of liposomes. The reaction samples were analysed by SDS–PAGE under non-reducing conditions in due time interval. As shown in Fig. 3f,g, the Atg3-Atg8 thioester intermediate was formed in almost the same speed for K19-K48-acetylated Atg3 and WT Atg3. The rate for formation of the Atg3-Atg8 thioester was faster than that for the Atg8-PE conjugate, which implied that the formation of the Atg3-Atg8 thioester intermediate was not the rate-determining step in Atg8 lipidation^26^.

### Effect of acetylation on Atg3 and liposome interaction

Ruling out the effect of acetylation on regulating Atg3-Atg8 interaction, we turned to other factors for identification of the driving force leading to the enhanced Atg8-PE formation. We noticed an interesting recent finding of Melia *et al*. that the N-terminal motif of mouse Atg3 (a homologue of yeast Atg3) could fold into an amphipathic helix to sense the hydrophobicity and curvature of membranes. Melia *et al*. showed that the membrane-binding property of this helix could be regulated by some amino-acid modification in its sequence (for example, Lys to Val mutation)^27^. We thus came up with a hypothesis that side-chain Lys acetylation of Atg3 was the physiologically relevant modification of the amphipathic helix to regulate the membrane-binding affinity.

**Figure 4:**
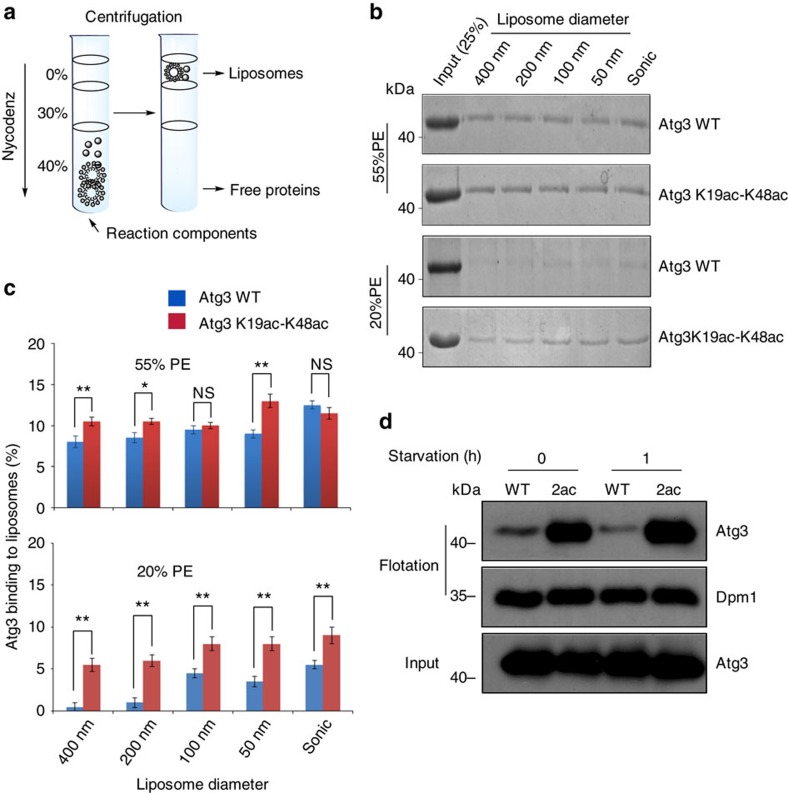
Interaction between Atg3 and membrane. (**a**) A schematic of flotation assay. Only lipidated protein can be recovered at the top of the gradient. (**b**) WT Atg3 and K19-K48-diacetylated Atg3 were incubated with liposomes containing 20 or 55% PE. The top fractions were analysed by SDS–PAGE. (**c**) Quantification of liposome-associated Atg3. The graph shows results of three independent experiments and error bars represent s.d. *P* values mean the comparison with Atg3 WT on the same liposome size and PE concentration. The panels show results of three independent experiments and error bars represent s.d. **P<0.01, *P<0.05; NS, not significant (Student's *t*-test). (**d**) ER membranes were isolated from the yeast cells, which were cultured in SD (−N) for 0 and 1 h. An aliquot of 1 μM Atg3 WT or Atg3 K19ac-K48ac was incubated with ER membranes at 30 °C for 60 min. Then the mixtures were subjected to the flotation assay. ER membrane-associated Atg3 was analysed by means of western blot by using an antibody to Atg3 and Dpm1 (an ER marker).

To examine the above hypothesis, WT or diacetylated Atg3 was incubated with PE-containing liposomes of different diameters and subjected to the flotation assay^28^. In this process, only liposome-associated Atg3 can be recovered by centrifugation using a non-equilibrium density gradient flotation (Fig. 4a). Two types of liposome systems, that is, the non-physiological high-PE density (55%) liposome usually used for mimicking Atg8 lipidation and the near-physiological low-PE density (20%) liposome, were used to measure the binding between acetylated Atg3 and liposomes^29^. In the 55% PE-containing liposome system, we found that the recovered Atg3 K19ac-K48ac had only moderate increase than Atg3 WT, showing that acetylation slightly improved the membrane binding. However, when the near-physiological 20% PE system was used, there was almost no Atg3 WT binding to liposome when 400 and 200 nm liposomes were used. Even though in the highly curvatured liposomes, we found that the recovered Atg3 K19ac-K48ac was two–three-fold more than Atg3 WT, showing that acetylation indeed improved the membrane binding (Fig. 4b,c).

We further performed Atg8 lipidation with differently sized liposomes between WT Atg3 and acetylated Atg3 (Supplementary Fig. 10). In 55% PE-containing liposome system, the formation of Atg8-PE showed similar speeds for both WT Atg3 and acetylated Atg3, regardless of the liposomes size. On the other hand, when 20% PE-containing liposomes were used, Atg8-PE formation in acetylated Atg3 reaction system was significantly faster than that with WT Atg3. Note that the speed of Atg8-PE formation depends on the size of liposome in acetylated Atg3 reaction system. When the size of liposome is smaller, Atg8-PE formation becomes faster. Taken together, we conclude that K19/K48 acetylation should promote autophagy through enabling Atg3 to bind physiological level of PE in a liposome-size-dependent manner.

### Effect of acetylation on Atg3 and ER membrane binding

Autophagosomes are formed from Omegasome, a subdomain of endoplasmic reticulum (ER) and ER-Golgi intermediate compartments have been identified as one of the membrane source for LC3 lipidation^30^. To investigate whether acetylation can enhance the binding between Atg3 and native membrane, we isolated ER membranes from yeast spheroplasts. We mixed Atg3 WT or Atg3 K19ac-K48ac with the ER membrane fraction and subjected the mixtures to flotation assay. The ER membrane-associated Atg3 was recovered by non-equilibrium density gradient centrifugation and analysed by western blot. As shown in Fig. 4d, K19-K48 acetylation dramatically enhanced the interaction between Atg3 and ER membrane, which implies that K19-K48 acetylation may promote Atg8 lipidation through enhancing the recruitment of Atg3 to membrane source for lipidation.

## Discussion

Protein acetylation plays an important role in regulating autophagy and Atg3 has been found to be acetylated when cells suffer from starvation. However, the role of Atg3 acetylation in regulating Atg8 lipidation remains unclear. To illustrate the mechanism, we efficiently obtained the homogeneous full-length Atg3 K19ac-K48ac via protein semi-synthesis for the first time. Using semi-synthesized Atg3 K19ac-K48ac, we carried out in depth mechanistic study, which revealed that acetylation markedly enhances the binding between Atg3 and membrane, and sequentially, the Atg8 lipidation.

**Figure 5:**
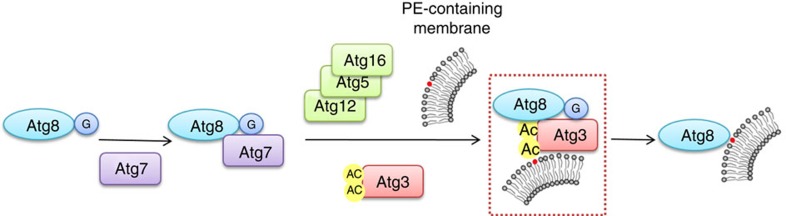
A model of acetylation of Atg3 functions in Atg8-PE conjugates. K19-K48-acetylated Atg3 might act as an adaptor protein to recruit Atg8 near lipids, thus enhanced the formation of Atg8-PE.

It is worth noting while acetylation enhances the interaction between Atg3 and Atg8 *in vivo*, it does not enhance Atg3/Atg8 interaction *in vitro*. The facts that semi-synthesized Atg3 K19ac-K48ac can pull down more Atg8 from cell lysate than WT Atg3, and semi-synthesized Atg3 K19ac-K48ac and Atg3 WT have similar binding affinity with recombinant Atg8, indicate Atg8 from cell lysate binds acetylated Atg3 more strongly than recombinant Atg8 purified from *E. coli.* At this point, we do not know what is the underneath mechanism, but we speculate that modifications or additional binding partner on Atg8 may explain this difference. Since Atg8 and PE-containing membrane are two substrates for lipidation reaction, this observation implies that besides being an E2 enzyme, acetylated Atg3 may also work as a tethering factor, which brings two substrates together, thus facilitating the lipidation reaction (Fig. 5).

Promotion of the interaction between Atg3 and PE-containing liposome by acetylation is consistent with several previous studies. First, the N-terminal seven amino acids of yeast Atg3 are involved in membrane binding^31^. Second, Atg3 serve as a membrane curvature sensor that can regulate Atg8 lipidation through N-terminal amphipathic helix interacting of Atg3 (ref. 27). Because amphipathic helices interact with the membrane interior by hydrophobicity and with the polar lipid head groups by electrostatic interactions, the change of hydrophobicity in this domain can promote or inhibit the lipidation process *in vitro*. Therefore, acetyl group of Atg3 may increase the hydrophobicity or enhance the electrostatic interactions to promote Atg3 membrane binding.

Membrane-bound acetylated Atg3 can possibly act as an adaptor protein to recruit Atg8 near lipids, thus enhanced the synthesis of membrane-anchored Atg8-PE. This presents a new theoretical model for the role of lysine acetylation in autophagy. Our finding exemplifies a new type of mechanism for protein acetylation, which we speculate may emerge as an important regulation mechanism for protein–lipid interaction. Our work further illustrates the power of protein semi-synthetic strategy in investigating mechanism of protein modification.

## Methods

### Peptide synthesis

All peptides used in this work were obtained from the Fmoc-based solid phase peptide synthesis. 2-Cl-(Trt)-Cl resin was treated with 5% NH_2_NH_2_ in N, N-Dimethylformamide (DMF, 30 min for twice) to prepare 2-Cl-(Trt)-NHNH_2_ resin. For each step of amino-acid coupling, the resin was coupled with a pre-activated solution of protected amino acids (4 equiv.), of HATU (3.8 equiv.), the DIPEA (8 equiv.) was successively dissolved in DMF solvent. The coupling time should extend from 1 to 2 h in a constant temperature shaker at 30 °C until ninhydrin test showed no bare amino residue was left on resin, then the resin was washed with DMF, dichloromethane (DCM), DMF for five times alternately. To remove the Fmoc group, 20% piperidine in DMF was added to the resins for 15 min (twice: 5 min and 10 min, respectively), then the resin was washed with DMF, DCM, DMF for five times alternately. After the de-protection of the Fmoc of the final amino acid, cleavage reagent (Typically a trifluoroacetic acid (TFA) cocktail of TFA/phenol/water/TIPS (88/5/5/2)) was added to the dry resin pre-washed with DCM. After 2 h, the resin was washed with an equal volume of TFA once. Combined washing liquids were concentrated by blowing with N_2_. The crude peptides were obtained by precipitating with cold ether and centrifugation at 5,000 r.p.m. The crude peptides were dissolved in 0.1% TFA containing co-solvent of acetonitrile and water, analysed by analytical HPLC plus ESI-MS and purified by semi-preparative HPLC and lyophilized immediately.

### Protein expression and purification

For protein expression and purification, Atg3(S54C-310) was prepared by the use of pET28a-SUMO protein expression system. *E. coli* BL21 (DE3) cells containing the vector pET28a-SUMO-Atg3(S54C-310) were grown in LB medium containing antibiotics (100 μg ml^−1^) with shaking overnight at 37 °C. After 1:100 dilution in LB medium containing antibiotics (100 μg ml^−1^), the culture was grown at 37 °C to an OD_600_ ∼0.6–0.8. Then, protein expression was induced by the addition of IPTG to the final concentration of 0.2 mM. After expression for 12–16 h at 16 °C, cells were collected by centrifugation (6,000 r.p.m., 10 min), and re-suspended in lysis buffer (20 mM Tris-HCl, 300 mM NaCl, 2 mM β-mercaptoethanol, pH 8.0). Bacterial lysate after sonication was loaded onto a Ni-NTA column (Histrap 5 ml, GE Healthcare). The column was washed with 50 ml washing buffer (20 mM Tris-HCl, 300 mM NaCl, 2 mM β-mercaptoethanol, pH 8.0 with 20 mM imidazole) and then eluted with elution buffer (20 mM Tris-HCl, 300 mM NaCl, 2 mM β-mercaptoethanol, pH 8.0 with 250 mM imidazole). The cleavage of the His-SUMO-Atg3(S54C-310) by using SUMO protease was performed under dialysis conditions to minimize thiazolidine formation. The elution buffer containing His-SUMO-Atg3(S54C-310) protein and SUMO protease were added to dialysis bag. The dialysis buffer contains 20 mM Tris-HCl, 300 mM NaCl, 5 mM β-mercaptoethanol, pH 8.0. The reaction was gently stirred and incubated at room temperature for 4 h. The cleavage reaction was analysed by SDS–PAGE and HPLC. Atg3(S54C-310) was further purified by Semi-preparative HPLC.

WT Atg3, Atg7 and Atg8 were prepared as follows. The region coding WT Atg3, Atg7 and Atg8 were inserted into pGEX-6 P-1 and expressed in *E. coli* BL21 (DE3) cells. Cells were grown in LB medium containing antibiotics (100 μg ml^−1^) with shaking overnight at 37 °C. After 1:100 dilution in LB medium containing antibiotics (100 μg ml^−1^), the culture was grown at 37 °C to an OD_600_ ∼0.6–0.8. Then, protein expression was induced by the addition of IPTG to the final concentration of 0.2 mM. After expression for 12–16 h at 16 °C, cells were collected by centrifugation (6,000 r.p.m., 10 min), and re-suspended in lysis buffer (20 mM Tris-HCl, 150 mM NaCl, 1 mM DTT, pH 8.0). After cell lysis, GST-fused Atg3, Atg7 and Atg8 were purified by affinity chromatography using a glutathione-Sepharose 4B column, and GST tag was excised with PreScission protease. WT Atg3, Atg7 and Atg8 were further purified by a Mono Q column followed by a Superdex 200 column.

### Expressed protein ligation

(A) Ligation of first and second segment of peptide hydrazide^32^. Peptide 1 (1.0 equiv.) was dissolved in 0.2 M phosphate buffer (pH 3.0) containing 6 M Gn·HCl. 1 M NaNO_2_ aqueous solution (5.0 equiv.) was added drop-wise and stirred for 20 min, at −12 °C. After the peptide hydrazide oxidizing to peptide azide, MPAA (50 equiv.) was added to the reaction buffer. The reaction was then removed to the room temperature and kept for 5 min to ensure a complete conversion of peptide thioester. Next, peptide 2 (1.2 equiv.) was added and the reaction buffer was adjusted to pH 6.5 with 2 M NaOH buffer slowly to initiate native chemical ligation. The reaction mixture was stirred at room temperature for 3 h. The reaction was monitored by RP-HPLC. After the ligation completed, the product 3 was isolated by semi-preparative HPLC with isolated yield of 67%. The reaction mixture was reduced by TCEP (30 mM, pH 7.0) before analysis and isolation. ESI-MS was used for molecular weight identification. (B) Ligation of segment 3 and 6. For the semi-synthesis of full-length acetylated Atg3, segment 6 (1.0 equiv.) and the first ligation product 3 (2.0 equiv.) were dissolved in 0.2 M phosphate buffer (pH 3.0) containing 6 M Gn·HCl. Then, 1 M NaNO_2_ aqueous solution (5.0 equiv.) was added drop-wise and stirred for 20 min, at −12 °C. After that, MPAA (50 equiv.) was added and the mixed solution was adjusted to pH 6.5 with NaOH (2.0 M) slowly. The reaction mixture was stirred at room temperature for 5 h. The reaction was monitored by RP-HPLC. After the ligation completed, the product 7 was isolated by semi-preparative HPLC with isolated yield of 55%.

### Protein folding

Purified Atg3 K19ac-K48ac, His_6_-Atg3 K19ac-K48ac and semi-synthetic Atg3 WT were dissolved in denaturation buffer (20 mM Tris, pH 7.5, 500 mM NaCl, 10 mM DTT, 5% glycerol, 8 M urea). The solutions were gently stirred by gradient dialysis against decreasing urea concentration from 8 to 0 M at 4 °C. Centrifuge the dialysed solution containing the renatured protein at 15,000*g* and 4 °C for 30 min to remove any remaining impurities or incorrectly folded protein, which is again aggregated. The supernatant of refolded protein was collected and further purified by a Superdex 200 column.

### CD spectroscopy

CD spectra were recorded on a Pistar π-180 spectrometer from 260 to 190 nm at 25 °C in a quartz cell with 1 mm path length. Each protein sample was dissolved to a final concentration of 0.2 mg ml^−1^ in 10 mM PBS (pH 7.5). The spectra were performed in triplicate, averaged, subtracted from blank and smoothed.

### Liposome preparation

All of the chloroform solutions of lipids were purchased from Avanti Polar Lipids. Liposomes used in this study were composed of 1-palmitoyl-2-oleoyl-*sn*-glycero-3-phosphocholine (POPC), 1,2-Dioleoyl-*sn*-Glycero-3-Phosphoethanolamine (DOPE) and phosphatidylinositol (bIPI) in a molar ratio of 35:55:10 or 70:20:10. All lipids were mixed in a glass vial and chloroform was evaporated under nitrogen air-flow to leave lipid films. The samples were further dried at 37 °C for 1 h. The lipid films were then re-suspended in Tris-HCl buffer (20 mM Tris, at pH 7.5, 150 mM NaCl and 1 mM MgCl_2_) to 4 mM. The resulting suspension of multi-lamellar liposomes was subjected to 20 cycles of snap freezing in liquid nitrogen and thawing in 42 °C water bath. The re-suspended lipids were then extruded through a different pore sizes (400, 200, 100 and 50 nm) polycarbonate filter using a hand-operated extruder (Avanti Polar Lipids) at room temperature and stored on ice at 4 °C.

### *In vitro* Atg8-PE conjugation reaction

The purified Atg7 (1 μM), Atg3 (1 μM) (WT Atg3 or semi-synthetic Atg3 K19ac-K48ac) and Atg8 (10 μM) were mixed with liposomes (350 μM lipids) in the presence of 1 mM DTT and 1 mM ATP in Tris-HCl buffer and incubated at 30 °C for 80 min. The reaction was stopped by 5 × SDS–PAGE sample buffer and boiled at 95 °C for 5 min. Samples were separated by urea SDS–PAGE and proteins were identified by Coomassie Brilliant Blue staining.

### *In vitro* pull-down assay

The BY4741 cell lysates or purified His_6_-Atg8 was mixed with purified WT Atg3 or semi-synthetic Atg3 K19ac-K48ac in Tris-HCl buffer and incubated at 25 °C for 1 h. Next, Ni-NTA resin was added to the mixture followed by further incubated at 25 °C for 1 h. After washing the beads with Tris-HCl buffer with 0.1% Tween-20 three times, bound proteins were eluted with elution buffer containing 250 mM imidazole, 20 mM Tris, at pH 7.5, 150 mM NaCl and 1 mM MgCl_2_. The eluted proteins were subjected to SDS–PAGE and protein bands were detected by Coomassie Brilliant Blue staining.

### SPR experiments

The binding affinities between Atg3 and Atg8 was measured by SPR (Biacore T200, GE Healthcare, Sweden), all at 25 °C. The purified WT Atg3 or semi-synthetic Atg3 (K19ac-K48ac) was diluted to a protein concentration of 40 mg ml^−1^ using 10 mM sodium acetate buffer (pH 4.5, GE Healthcare). The diluted Atg3 was immobilized by amine coupling to the surface of a CM 5 sensor chip (GE Healthcare). The target Atg3 immobilization levels were 400 RU. The CM 5 ship contained four flow cells: WT Atg3 and semi-synthetic Atg3 (K19ac-K48ac) were immobilized on flow cell 2 and 4, respectively, and another two flow cells were used as reference for non-specific binding. The purified Atg8 was injected over Atg3-immobilized CM 5 chip at 30 μl min^−1^ in running buffer (10 mM phosphate buffer with 2.7 mM KCl, 137 mM NaCl, and 0.05% surfactant P20, final pH 7.4, GE Healthcare) with 12 twofold dilutions at the lowest concentration, increasing to 3,200 nM. The contact and dissociation time were 30 s and 60 s, respectively. The surface of the sensor chip was regenerated by injecting 10 mM Glycine 3.0 (GE Healthcare) at a flow rate of 30 μl min^−1^ for 20 s for completely remove the tightly bound residual Atg8. Response units (RU) values were collected and the dissociation constant (*K*_D_) was calculated using BIAEvaluation Software, version 2.0. The buffer blanks were subtracted from each curve.

### MST experiments

Binding of different Atg3-Atg8 was also measured by MST analysis. Measurements were performed in standard treated capillaries on a Monolith NT. 115 instrument (20% LED, 20% MST power). Atg8 fluorescence label was performed with reactive dyes using N-Hydroxysuccinimide (NHS)–ester chemistry by using the Monolith NT. 115 Protein Labeling Kit RED, which reacts efficiently with the primary amines of proteins to form highly stable dye–protein conjugates. An aliquot of 6 μl Dye NT-647 NHS in 100% DMSO was added into 200 μl 10 μM Atg8 (buffer: 50 mM Tris-HCl pH 7.4, 150 mM NaCl, 10 mM MgCl_2_, 0.05% Tween-20), then the mixture was incubated in dark at 4 °C for 30 min. The labelled Atg8 was purified by gel-filtration column B in the kit. The labelled Atg8 was incubated for 5 min at room temperature in the dark with different concentrations of WT Atg3 or semi-synthetic acetylated Atg3 and 3–5 μl of samples were loaded into standard glass capillaries for analysis. Nano Temper Analysis software was used to fit the data and determine the apparent *K*_D_ values. The *K*_D_ value is the mean of three independent experiments.

### *In vitro* assay of Atg3-Atg8 thioester formation

The purified Atg7 (1 μM), Atg3 (1 μM) (expressed Atg3 WT, semi-synthetic Atg3 WT or semi-synthetic Atg3 K19ac-K48ac) and Atg8 (10 μM) were mixed in the presence of 0.2 mM DTT and 1 mM ATP in Tris-HCl buffer and incubated at 30 °C for 15 min. The reaction was stopped by SDS sample buffer (non-reducing) and boiled at 42 °C for 5 min. Samples were separated by NuPAGE 12% Bis-Tris gels (Invitrogen) and proteins were identified by Coomassie Brilliant Blue staining.

### *In vitro* Atg3-binding reaction and flotation assay

WT Atg3 (expressed or semi-synthetic) or semi-synthetic Atg3 K19ac-K48ac (10 μM) was incubated with liposomes containing 20 or 55% DOPE (1 mM lipids) in Tris-HCl buffer at 30 °C for 90 min in a total volume of 300 μl. The mixtures were adjusted to 40% (w/v) Nycodenz by adding with 300 μl 80% (w/v) Nycodenz in Tris-HCl buffer. The resulting mixture was overlaid with 1300 μl Tris-HCl buffer containing 30% (w/v) Nycodenz and 100 μl Tris-HCl buffer without Nycodenz. The samples were centrifuged at 200,000*g* in a TCL-55 rotor (Beckman) for 4 h. The liposomes and lipid-anchored proteins were collected from the top 100 μl of the mixtures and analysed by SDS–PAGE. Protein bands were detected by Coomassie Brilliant Blue staining and band intensities on SDS–PAGE were quantified by using Image software on ChemDocXRS+ (Bio-Rad). Student's *t-*test was used to calculate *P* values and evaluate the significance of difference between recovered Atg3 WT and Atg3 K19ac-K48ac.

### Isolation of ER

ER membranes were isolated from yeast spheroplasts using sucrose step gradient as described previously^33^. The isolation yield was about 7 mg of protein per 5,000 OD_600_ units of cells.

### Data availability

The data that support the conclusions of this study are available from the corresponding author on request.

## Additional information

**How to cite this article:** Li, Y.-T. *et al*. A semisynthetic Atg3 reveals that acetylation promotes Atg3 membrane binding and Atg8 lipidation. *Nat. Commun.* **8,** 14846 doi: 10.1038/ncomms14846 (2017).

**Publisher's note:** Springer Nature remains neutral with regard to jurisdictional claims in published maps and institutional affiliations.

## Supplementary Material

Supplementary Information Supplementary figures and supplementary methods.
