# Supplementary material for: A semisynthetic Atg3 reveals that acetylation promotes Atg3 membrane binding and Atg8 lipidation
Source: Nat Commun. 2017 Mar 22;8:14846. doi: 10.1038/ncomms14846 (PMC5473643; doi:10.1038/ncomms14846)
Supplement: Supplementary Information — Supplementary figures and supplementary methods. [file ncomms14846-s1.pdf]

Supplementary Methods

Materials

2-Chlorotrityl resins were purchased from Hecheng Technology (Tianjing, China). Fmoc-amino acids including Fmoc-Acetyl-L-Lys were purchased from CS Bio,GL Biochem (Shanghai,China). *O*-(6-Chlorobenzotriazol-1-yl) -*N,N,N',N'*-tetramethyluroniumhexafluorophosphate (HCTU), *O*-(7-azabenzotriazol-1-yl) -*N,N,N',N'*-tetramethyluroniumhexafluorophosphate (HATU), 1-Hydroxy-7-azabenzotriazole (HOAt) and 1-hydroxy-benzotriazole (HOBt) were purchased from GL Biochem (Shanghai, China). *N,N'*-Diisopropyl-carbodiimide (DIC), 1,2-ethanedithiol, N,N-diisopropylethylamine (DIPEA), triisopropylsilane (TIPS) and 4-mercaptophenylacetic acid (MPAA) were purchased from Ouhe Technology (Beijing, China). Dithiothreitol (DTT) was purchased from Aladdin (Shanghai, China). Acetonitrile (HPLC grade) was purchased from J. T. Baker (Phillipsburg, NJ, USA). $Na_2HPO_4{\cdot}12H_2O$, CH_3OH, guanidine hydrochloride (Gn·HCl), Et_2O, hydrazine hydrate 85% and N,N-Dimethylformamide (DMF) were purchased from Sinopharm Chemical Reagent. Thioanisole and trifluoroacetic acid (TFA) (HPLC grade) were purchased from J&K Scientific (Beijing, China). Cyanine 5.5-OSu was purchased from HEOWNS (Tianjin, China). NaOH, $NaHCO_3$, CH_2Cl_2 (DCM), acetic acid, hydrochloric acid and $NaNO_2$ were purchased from Beijing Chemical Works (Beijing, China). CM5 chips and phosphate buffer solution (10×) was purchased from GE healthcare.

HPLC and FPLC

Analytical RP-HPLC was run on a SHIMADZU (Prominence LC-20AT) instrument using an analytical column (Grace Vydac C4, 250 ×4.6 mm, and C18, 250 ×4.6 mm, 5 µm particle size, flow rate 1.0 mL/min, rt). Analytical injections were monitored at 214 nm and 254 nm. Semi-preparative HPLC was run on a SHIMADZU (Prominence LC-20AT) instrument using a semi preparative column (Grace VydacC4, 250 ×10 mm,and C8, 250 ×10 mm,10 µm particle size, flow rate 3.0 mL/min). Solvent A was 0.1% TFA in acetonitrile, and solvent B was 0.1% TFA in water. Both solvents were filtered through 0.22 µm filter paper and sonicated for 30 min before use.

FPLC was run on a GE Healthcare (AKTA purifier 10 UPC-F920) instrument using a Superdex 200 column or Mono Q column. The injections were monitored at 280 nm. All the buffers were filtered through 0.22 µm filter paper and sonicated for 30 min before use.

Molecular biology and biochemistry

Primers were ordered from Biomed Biotech (Beijing) Co., Ltd. All enzymes were ordered from New England Biolabs (NEB). Bacterial cells were grown in LB (Luria-Bertani) broth or on LBagar medium (Sigma). All pictures of protein gels were taken on ChemDocXRS+ (Bio-Rad).

For SDS-PAGE, samples were loaded onto 12-15% SDS-PAGE gels and electrophoresed for 30 min at 80 V and 50 min at 150 V.

For urea-SDS-PAGE, samples were loaded onto 15% urea-SDS-PAGE gels and electrophoresed for 80 min at 25 mA.

For NuPAGE, samples were loaded onto 12% NuPAGEBis-Trisgels and electrophoresed for 30 min at 200 V.

Mass spectrometry

High-resolution ESI mass spectra were measured on Agilent 6210 Time of Flight Mass Spectrometer. Normal ESI mass spectra were measured on a Bruker Daltonics DataAnalysis 3.0 workstation.MALDI-TOF mass spectra were measured on an Applied Biosystems 4700 Proteomics Analyzer 283. A solution of 10 mg/mL matrix α-cyano-4-hydroxy cinnamic acid containing 1:1 v/v (0.1% TFA in acetonitrile / 0.1% TFA in water) was used for generating the probe-matrix mixture.

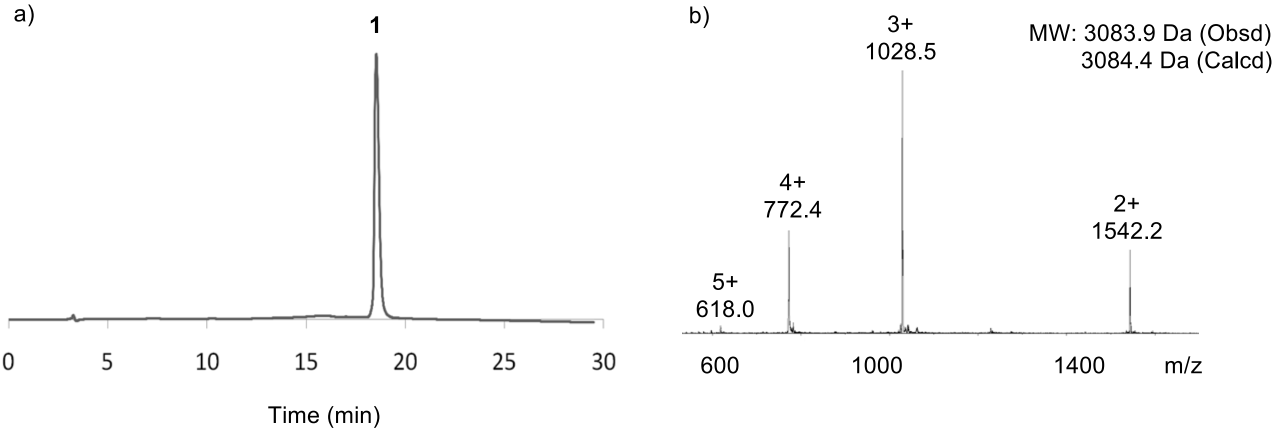

Supplementary Figure 1. Characterization of peptide **1** a) analytical HPLC chromatograms (λ = 214 nm) of purified peptide **1**. HPLC conditions: a liner gradient of 20-80% acetonitrile (containing 0.08-0.1% TFA) in water (containing 0.08-0.1% TFA) over 30 min. b) ESI-MS observed 3083.9 Da, calculated 3084.4 Da.

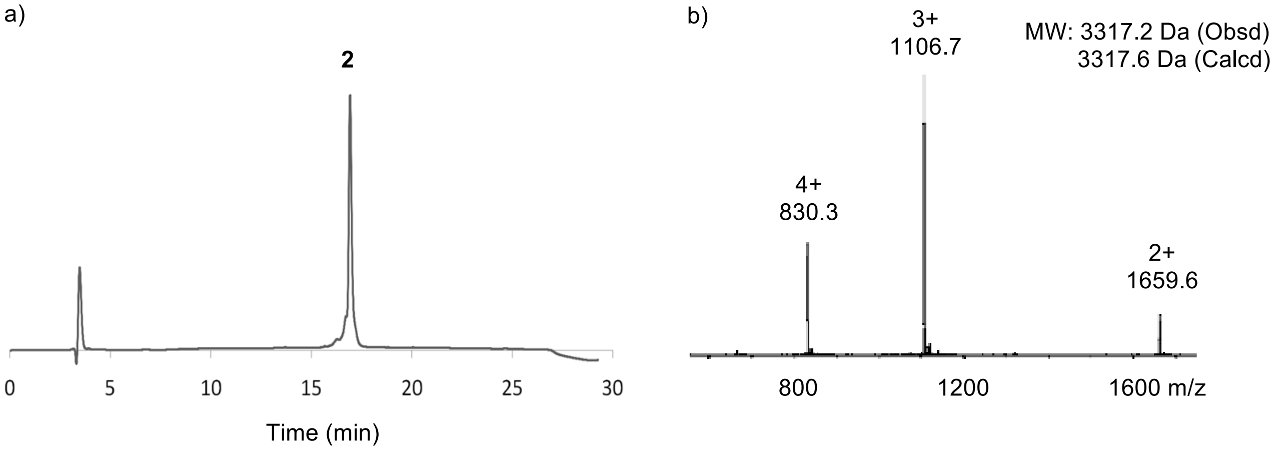

Supplementary Figure 2. Characterization of peptide 2 a) analytical HPLC chromatograms (λ = 214 nm) of purified peptide 2. HPLC conditions: a liner gradient of 20-70% acetonitrile (containing 0.08-0.1% TFA) in water (containing 0.08-0.1% TFA) over 30 min. b) ESI-MS observed 3317.2 Da, calculated 3317.6 Da.

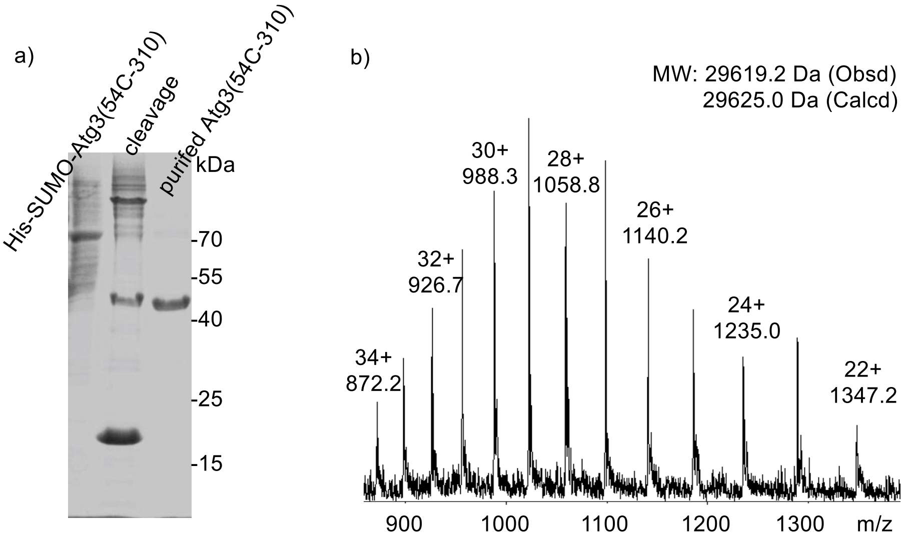

Supplementary Figure 3. Characterization of Atg3(S54C-310). a) SDS-PAGE data, (b) ESI-MS data, observed 29619.2 Da, calculated 29625.0 Da.

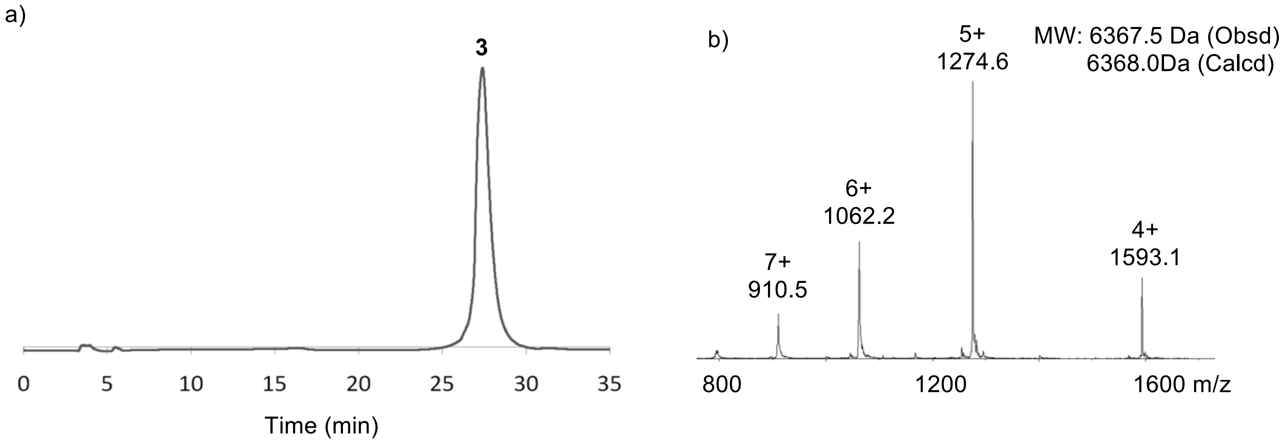

Supplementary Figure 4. Characterization of the first ligation product **3** a) analytical HPLC chromatograms (λ = 214 nm) of purified product **3**. HPLC conditions: a liner gradient of 20-65% acetonitrile (containing 0.08-0.1% TFA) in water (containing 0.08-0.1% TFA) over 35 min. b) ESI-MS observed 6367.5 Da, calculated 6368.0 Da.

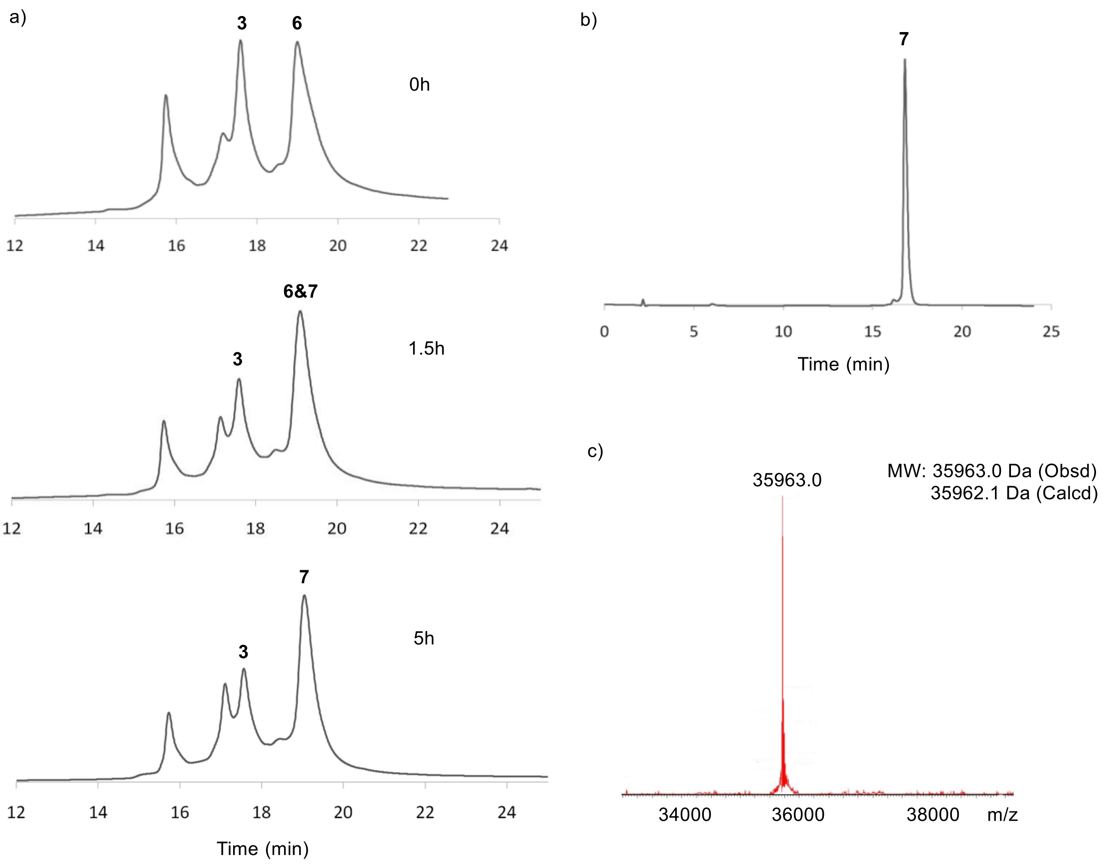

Supplementary Figure 5. Characterization of the full-length acetylated Atg3. a) Analytical HPLC traces of EPL of **3** and **6**. b) Analytical HPLC chromatograms analysis (λ = 214 nm) of purified product **7**. HPLC conditions: a liner gradient of 30-90% acetonitrile (containing 0.08-0.1% TFA) in water (containing 0.08-0.1% TFA) over 30 min. c) Q-TOF-MS observed 35963.0 Da, calculated 35962.1 Da.

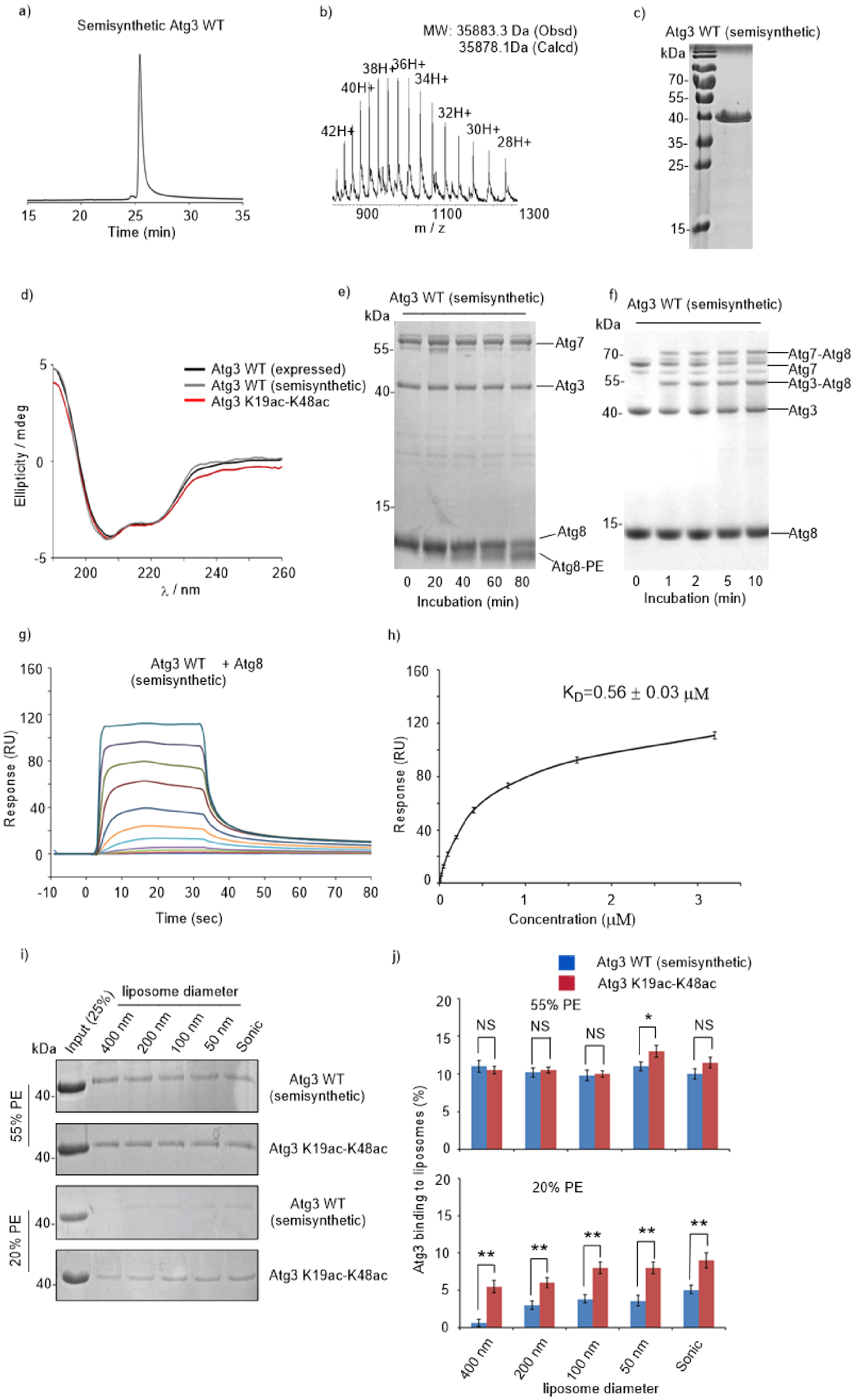
a)
Semisynthetic Atg3 WT
Time (min)
b)
MW: 35883.3 Da (Obsd)
35878.1Da (Calcd)
42H+
40H+
38H+
36H+
34H+
32H+
30H+
28H+
m / z
c)
Atg3 WT (semisynthetic)
kDa
d)
Atg3 WT (expressed)
Atg3 WT (semisynthetic)
Atg3 K19ac-K48ac
Ellipticity / mdeg
λ / nm
e)
Atg3 WT (semisynthetic)
kDa
Atg7
Atg3
Atg8
Atg8-PE
Incubation (min)
f)
Atg3 WT (semisynthetic)
kDa
Atg7-Atg8
Atg7
Atg3-Atg8
Atg3
Atg8
Incubation (min)
g)
Atg3 WT + Atg8
(semisynthetic)
Response (RU)
Time (sec)
h)
K_D=0.56 ± 0.03 μM
Response (RU)
Concentration (μM)
i)
liposome diameter
kDa
Input (25%)
400 nm
200 nm
100 nm
50 nm
Sonic
55% PE
20% PE
Atg3 WT
(semisynthetic)
Atg3 K19ac-K48ac
j)
Atg3 WT (semisynthetic)
Atg3 K19ac-K48ac
55% PE
20% PE
NS
*
**
Atg3 binding to liposomes (%)
liposome diameter

Supplementary Figure 6. Characterization of the semisynthetic wild type Atg3 a) analytical HPLC chromatograms (λ = 214 nm) of purified Atg3 WT. HPLC conditions: a liner gradient of 30-90% acetonitrile (containing 0.08-0.1% TFA) in water (containing 0.08-0.1% TFA) over 35 min. b) ESI-MS observed 35883.3 Da, calculated 35878.1 Da. c)
SDS-PAGE analysis of semisynthetic Atg3 WT. d) Circular dichroism (CD) spectra of Atg3. e) *In vitro* reconstitution of Atg8 lipidation using semisynthetic Atg3 WT. f) Formation of semisynthetic wild type Atg3~Atg8 thioester intermediate was analyzed by SDS-PAGE. g-h) SPR binding study of semisynthetic Atg3 WT to Atg8.i) Atg3 binding to liposomes. Semisynthetic Atg3 WT and Atg3 K19ac-K48ac were incubated with liposomes containing 20% or 55% PE. SDS-PAGE samples were prepared as Figure 4b.j) Quantification of liposome-associated Atg3. The graphics shows the results of three independent experiments and error bars represent standard deviation.P values mean the comparison with Atg3 WT on the same liposome size and PE concentration. The panels show results of three independent experiments and error bars represent standard deviation. ***P*< 0.01, *P < 0.05; NS, not significant (Student's *t* test).

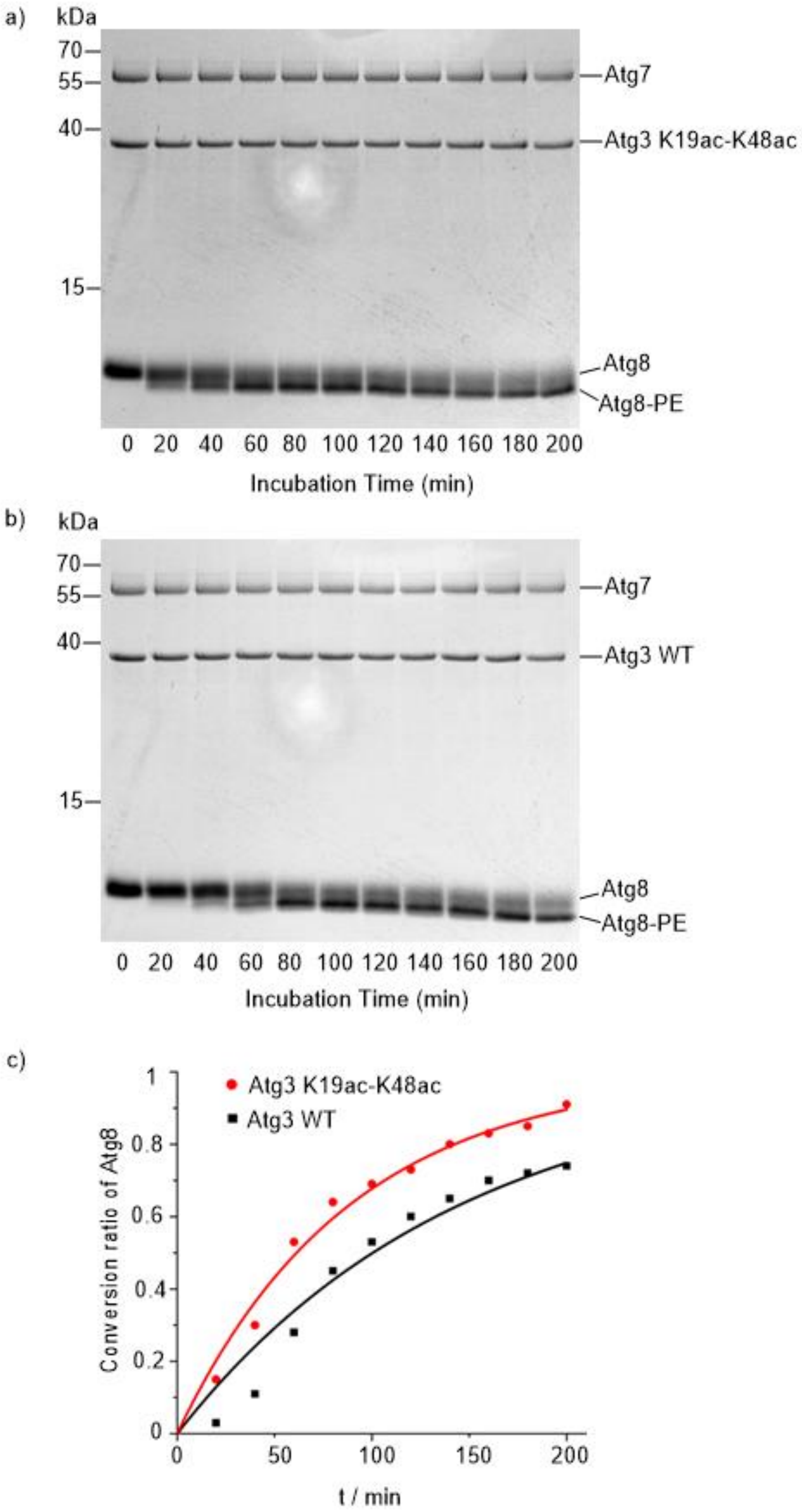

Supplementary Figure 7.a) and b) Atg8 lipidation reaction *in vitro*. SDS-PAGE and reaction samples prepared as described in Figure 2a.c) Relationship between reaction time t and conversion ratio of Atg8 ($1\text{-}C_0'/C_0$), C_0 represents the initial reaction concentration of Atg8, C_0' represents the concentration of Atg8 at different reaction time. According to pseudo-first-order reaction equation$C_0' = C_0e^{(-kt)}$, the reaction rate constant k of Atg3 WT was calculated to be 0.0063 min^{-1}, whereas the reaction rate constant of Atg3 K19ac-K48ac was 0.012 min^{-1}.

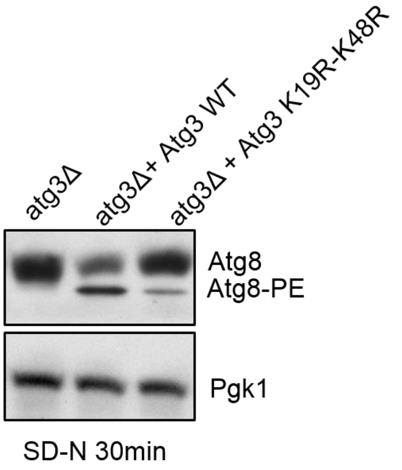

Supplementary Figure 8. Immunoblotting assayforthe comparingof Atg8–PE formation in yeast cells between WT Atg3 andAtg3 K19R-K48R mutants.*atg3*Δ, *atg3*Δ+Atg3 WTand *atg3*Δ+Atg3 K19R-K48R cells were grown to log growth phase, and subsequently were starved in nitrogen starvation for 30 min. Protein was extracted and subjected to urea-SDS-PAGE, followed by probed with indicated antibody. As shown in Supplementary Figure 8.Atg8-PE level was decreased in *atg3*Δ+Atg3 K19R-K48R cells in which Atg3 K19-K48 acetylation was suppressed.

a)

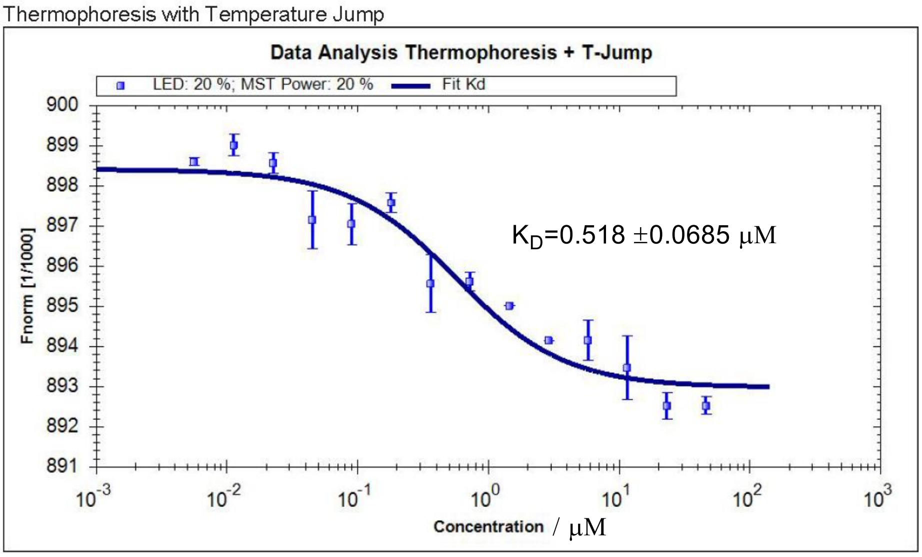

b)

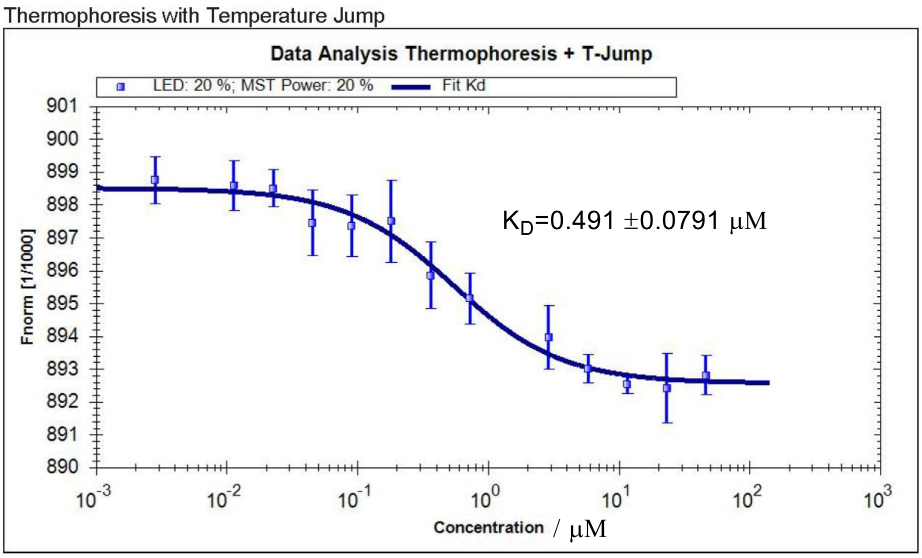

Supplementary Figure 9. a)MST binding measurements of Atg3 WT (expressed) to Atg8. b) MST binding measurements of semisynthetic Atg3 K19ac-K48acto Atg8. Theerror bars indicate the standard errors of the means from three independent experiments.

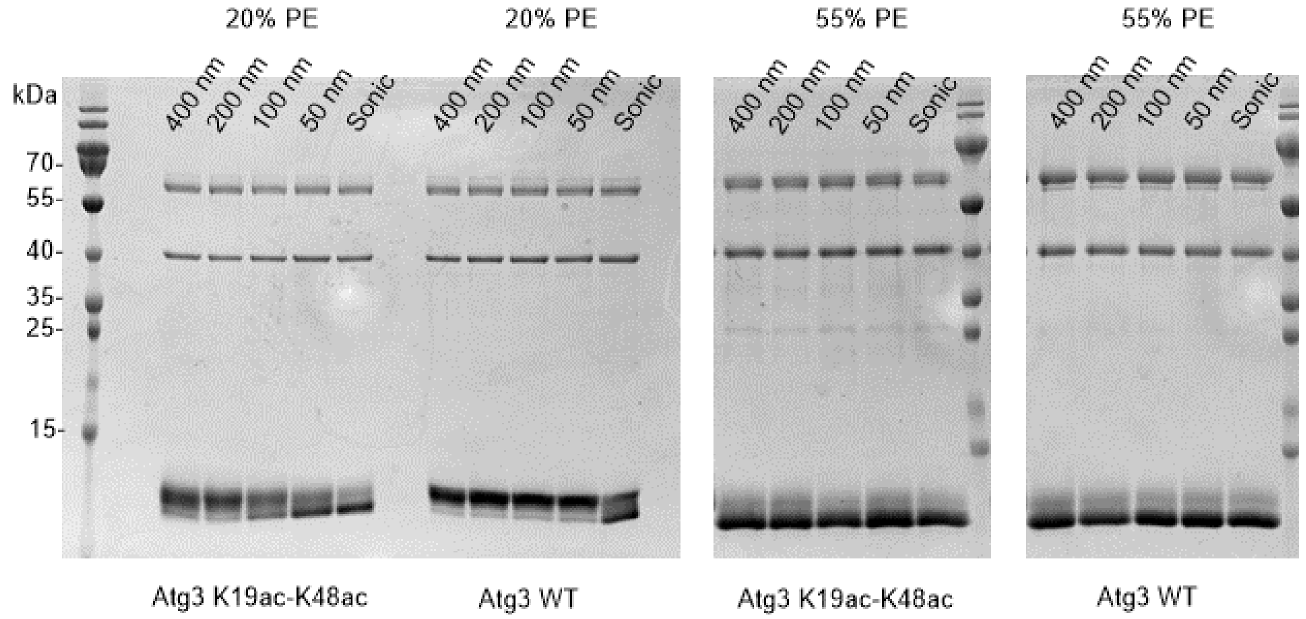

Supplementary Figure 10. Atg8 lipidation with differently-sized liposomes for wild type Atg3 and acetylated Atg3. The reaction mixtures of Atg7, Atg3 (acetylated or WT), Atg8 and liposomes were incubated at 30 ^{o}C for 40 min. The reaction was stopped with loading buffer, heated for 5 min at 95 ^{o}C, and then analyzed by SDS-PAGE.

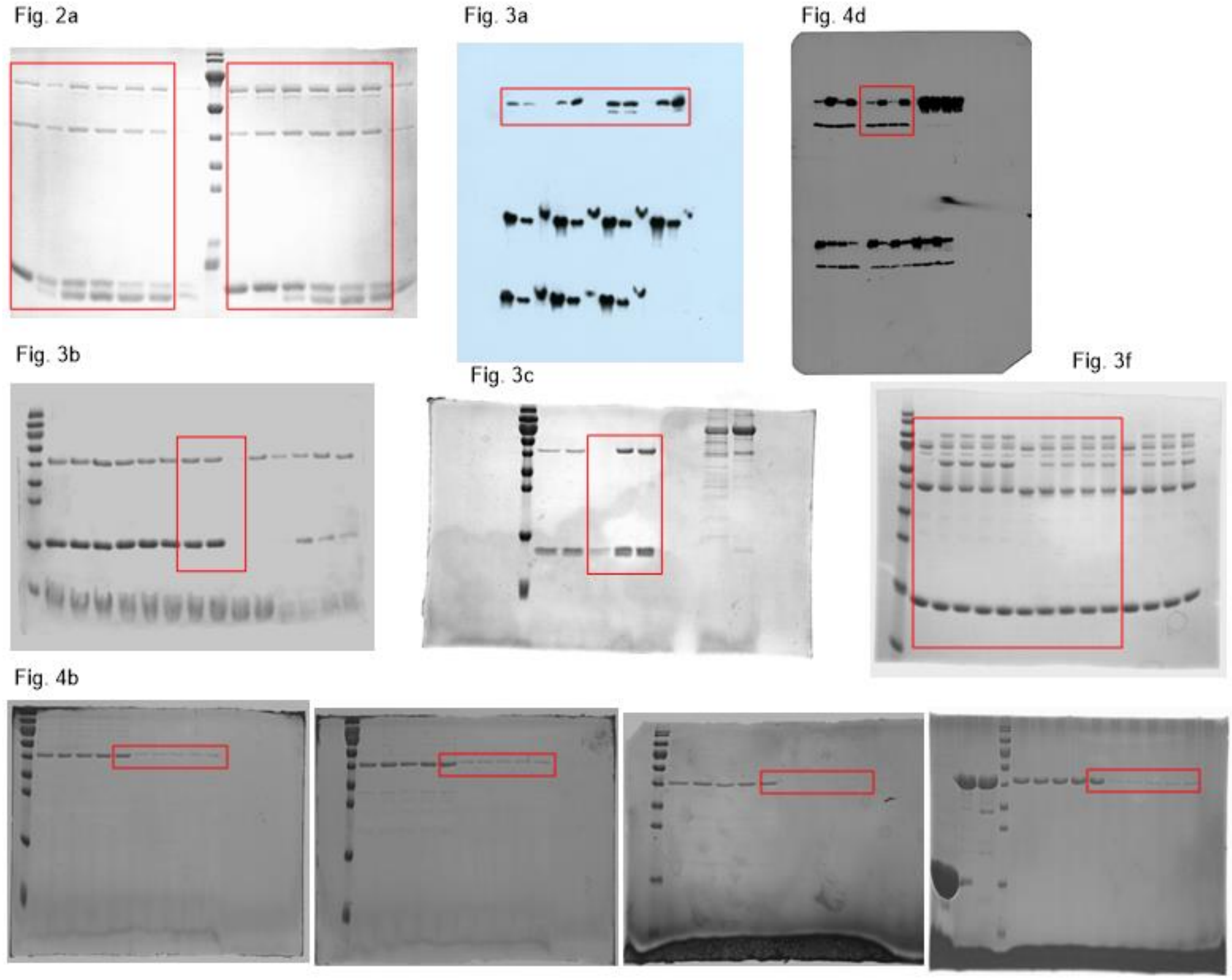

Supplementary Figure 11. Raw images used in the main text.
